# Factors associated with persistent anti-desmoglein positivity after remission in pemphigus vulgaris: a prospective registry-based cohort study

**DOI:** 10.3389/fimmu.2025.1557556

**Published:** 2025-05-13

**Authors:** Yuxi Zhou, Yiyi Wang, Mi Wang, Xingli Zhou, Limei Luo, Wei Yan, Wei Li

**Affiliations:** ^1^ Department of Dermatology & Venerology, Rare Diseases Center, West China Hospital, Sichuan University, Chengdu, China; ^2^ Department of Dermatology, Beijing Hospital, National Center of Gerontology, Institute of Geriatric Medicine, Chinese Academy of Medical Sciences & Peking Union Medical College, Beijing, China; ^3^ Department of Laboratory Medicine, West China Hospital of Sichuan University, Chengdu, Sichuan, China

**Keywords:** pemphigus vulgaris, desmoglein, antibody titers, clinical phenotype, disease activity, risk factors

## Abstract

**Introduction:**

Anti-desmoglein (Dsg) antibodies are well-established markers correlated with clinical phenotype and disease severity in pemphigus vulgaris (PV). However, elevated anti-Dsg antibody levels have been observed in some patients during clinical remission (CR). This study aimed to identify clinical characteristics and risk factors in PV patients with elevated anti-Dsg antibodies after achieving CR.

**Methods:**

We conducted a cohort study based on the prospective registry database of autoimmune bullous diseases patients at West China Hospital between April 2016 and March 2022. PV patients with at least 12 months of follow-up were enrolled. The pemphigus disease area index (PDAI) and anti-Dsg antibody titers were measured at baseline and 1, 3, 6, 9, 12 months during follow-up. Univariate, multivariate analyses and receiver operating characteristics (ROC) curves were performed to identify associated factors with persistent antibody positivity and optimal cut-off values respectively. The primary outcome was the persistent positivity of antibodies against Dsg after achieving CR.

**Results:**

Among 239 PV patients enrolled in this study, 118 (49%) achieved CR. Cataracts were identified as an independent risk factor for persistent anti-Dsg1 positivity after CR. Higher baseline anti-Dsg3 antibody titers and PDAI scores were significant predictors of increased anti-Dsg3 levels post-CR, with gender also being a contributing factor. ROC analysis determined a cut-off value of 157.4 U/mL for anti-Dsg3 with 56.3% sensitivity and 82.6% specificity.

**Conclusion:**

The presence of Cataracts may indicate persistent anti-Dsg1 positivity after CR, while elevated anti-Dsg3 titers and PDAI scores at baseline may predict sustained elevated anti-Dsg3 post-CR.

## 1 Introduction

Pemphigus is a group of life-threatening autoimmune disorders characterized by intraepidermal blisters and erosions affecting both the skin and mucous membranes (1). Pemphigus vulgaris (PV), the most prevalent form of pemphigus, has an annual incidence of 1–5 cases per million and is associated with a high mortality rate (2). The development of PV is mainly due to autoantibodies targeting desmosome adhesion molecules desmoglein (Dsg) 1 and 3, leading to loss of intercellular adhesion and subsequent acantholysis (3). The Dsg compensation theory explains the correlation between clinical phenotypes and autoantibody profiles (4), classifying PV into three subtypes: mucocutaneous PV (MCPV), mucosal-dominant PV (MDPV), and cutaneous PV (CPV). Furthermore, it is considered that anti-Dsg antibody levels generally correlate positively with disease activity (5–7), serving as a valuable marker for monitoring disease severity, particularly after achieving complete remission (CR).

However, previous studies have reported persistent anti-Dsg antibody positivity in some patients who have achieved remission (8–10). Our clinical observations align with these findings, indicating that anti-Dsg1 and anti-Dsg3 antibody detection does not always correlate with clinical phenotype or disease severity. This inconsistency suggests that additional factors influencing anti-Dsg antibody levels may have been overlooked, potentially providing new insights into the underlying pathogenesis of PV. To address this discrepancy, we conducted the cohort study to explore factors associated with persistent anti-Dsg1 and anti-Dsg3 antibodies positivity following remission and to evaluate their role in PV management.

## 2 Materials and methods

### 2.1 Data source, study design, and patient enrollment

The data collection and management for this cohort study was based on a prospective registry database of autoimmune bullous diseases at West China Hospital (AIBD-WCH). This database includes patients diagnosed with autoimmune bullous diseases at the Department of Dermatology, West China Hospital since April 1, 2016, and continuously gathers their clinical and laboratory data, which was approved by the ethics committee of West China Hospital of Sichuan University [approval number 2017(241)] and conducted in accordance with the principles of the Declaration of Helsinki. Written informed consent was obtained for all patients.

Based on the database, we included PV patients who visited our department between April 2016 and March 2022 with a follow-up duration of more than 12 months. Patients were eligible if they met the diagnostic criteria of PV according to international guideline indicators (11, 12). Those diagnosed with other types of pemphigus or with a follow up duration of less than 12 months were excluded.

### 2.2 Follow-up and data collection

After enrolling eligible patients into the cohort, we collected detailed baseline information, including sex, age, disease duration, lesions characteristics, disease severity, The pemphigus disease area index (PDAI) scores, anti-Dsg1 and anti-Dsg3 antibody titers, comorbidities, and treatment regimens. During follow-up visits at months 1, 3, 6, 9, and 12, assessments of disease severity, laboratory data collection, and disease status were conducted. Antibody titers against Dsg1 and Dsg3 were measured using Enzyme Linked Immunosorbent Assay (ELISA) (MBL, Nagoya, Japan), with cut-off values set at 20 U/mL for both. Disease activity in the skin and mucosa was evaluated using PDAI and classified as mild (<15), moderate (15–45), or severe (≥45) (13).

### 2.3 Definitions of disease status

In this study, the primary outcome was the persistent positivity of antibodies against Dsg1 and Dsg3 in PV patients after achieving CR. According to international consensus and established guidelines (14, 15), CR was defined as the absence of new lesions or resolution of existing lesions with the cessation of treatment or use of minimal therapy (prednisone ≤10 mg/day and/or minimal adjuvant therapy) for at least 2 months. Based on this, persistent anti-Dsg1 and anti-Dsg3 antibody positivity after CR was defined as anti-Dsg1 and anti-Dsg3 antibody titers remaining above 20 U/mL following the achievement of CR.

### 2.4 Statistical analysis

Baseline characteristics were presented as mean with standard deviation (SD) or median with interquartile range (IQR) of 25–75 depending on the normality of the data. Categorical variables were described using frequencies and percentages. Comparisons between categorical and continuous variables were performed using the chi-square test and t-test, respectively. Differences between groups were analyzed using the Mann-Whitney U test and the Kruskal-Wallis test. Spearman rank coefficient was used to assess the correlation of antibody titers (Dsg1, Dsg3) with PDAI and clinical outcomes. *P* < 0.05 was considered statistically significant. Odds ratio (OR) and 95% Confidence Interval (CI) were estimated using logistic regression. Receiver operating characteristic (ROC) analysis was conducted to determine the cut-off values for anti-Dsg antibody titers. Analyses were performed using SPSS Version 26.0 software (IBM Corporation).

## 3 Results

### 3.1 Demographic and clinical characteristics of patients

A total of 592 patients were enrolled in the AIBD-WCH registry between April 2016 and March 2022. Following inclusion and exclusion criteria, 239 eligible PV patients were included in this study. The cohort consisted of 110 male (46.0%) and 129 female (54.0%) patients, with a male: female ratio of 1:1.17. Their median age was 47 years (IQR: 38-55). Baseline demographic and clinical characteristics are detailed in **Table 1**.

**Table 1:** Characteristics of patients diagnosed with pemphigus vulgaris, stratified by complete remission status after 12-month follow up.

Characteristics	Overall population (n=239)	CR (n=118)	Non-CR (n=121)
Male, n (%)	110 (46)	52 (45)	58 (48)
Male: female	1:1.17	1:1.23	1:1.09
Age at diagnosis, median (IQR), year	47 (38-55)	48 (40-55)	47 (38-57)
Disease duration at baseline, Median (IQR), month	6 (3-12)	6 (3-12)	5 (3-12)
Clinical presentation at diagnosis, n (%)			
MDPV	43 (18)	20 (17)	23 (19)
MCPV	171 (72)	86 (73)	85 (70)
CPV	25 (10)	12 (10)	13 (11)
Dsg1 positivity, n (%)	179 (75)	84 (71)	95 (79)
Dsg3 positivity, n (%)	224 (94)	111 (94)	113 (93)
Anti-Dsg1 at baseline, Median (IQR)	82.4 (19.7-144.8)	87.3 (16.4-146.0)	80.1 (28.4-141.1)
Anti-Dsg3 at baseline, Median (IQR)	131.2 (81.2-166.2)	129.2 (73.8-160.7)	132.4 (83.1-169.2)
PDAI score at baseline, Median (IQR)	16 (6-28)	14 (6-27)	17 (7-29)
Disease severity			
Mild, n (%)	113 (47)	61 (52)	52 (8)
Moderate, n (%)	116 (49)	52 (44)	64 (53)
Severe, n (%)	10 (4)	5 (4)	5 (4)
Affected site			
Skin only, n (%)	18 (8)	8 (7)	10 (8)
Mucosa only, n (%)	38 (16)	17 (14)	22 (18)
Both skin and mucosa, n (%)	183 (76)	93 (79)	89 (74)
Comorbidities			
Cardiovascular, n (%)	16 (7)	7 (6)	9 (7)
Respiratory, n (%)	13 (5)	7 (6)	6 (5)
Gastroenteric, n (%)	28 (12)	16 (14)	12 (10)
Endocrine, n (%)	18 (8)	8 (7)	10 (8)
Hematological, n (%)	2 (1)	1 (1)	1 (1)
Neurological, n (%)	3 (1)	1 (1)	2 (2)
Urinary, n (%)	5 (2)	3 (3)	2 (2)
Treatment regimen			
GC, n (%)	55 (23)	28 (24)	27 (22)
GC combined with IS, n (%)	122 (51)	58 (49)	64 (53)
RTX, n (%)	62 (26)	32 (27)	30 (25)

CR, complete Remission; IQR, interquartile range; PDAI, pemphigus Disease Area Index; Dsg, desmoglein; MDPV, mucosal-dominant pemphigus vulgaris; MCPV, mucocutaneous pemphigus vulgaris; CPV, cutaneous pemphigus vulgaris; GC, Glucocorticoid; IS, Immunosuppressant; RTX, Rituximab.

Among the 239 PV patients, the MCPV subtype was the most prevalent, comprising 72% of the cohort, followed by MDPV at 18%, and CPV at 10%. This distribution indicates that most PV patients presented with both skin and mucosal involvement. Regarding autoantibody profiles, 75% of patients were positive for anti-Dsg1 antibodies, while 94% were positive for anti-Dsg3 antibodies, emphasizing the close association between Dsg3 autoantibodies and PV pathophysiology.

Among them, a total of 118 patients (49%) achieved CR after a 12-month follow-up, while the remaining 121 patients (51%) did not achieve CR (**Table 1**). Baseline characteristics, including age, disease duration, and antibody positivity rates, were similar between CR and non-CR groups.

Regarding treatment regimens, the combination of glucocorticoids (GC) and immunosuppressants (IS) was the most frequently used, prescribed to 51% of the total population, with 49% of patients who have achieved CR and 53% in the non-CR group receiving this treatment. Rituximab (RTX) was administered to 26% of the cohort, with a nearly equal distribution between the CR (27%) and non-CR (25%) groups. There were no statistically significant differences in the anti-Dsg1 and anti-Dsg3 positivity between patients treated with GC combined with IS and those treated with RTX (*P >*0.05).

### 3.2 Antibody titers and disease severity during follow-up

**Figure 1:**
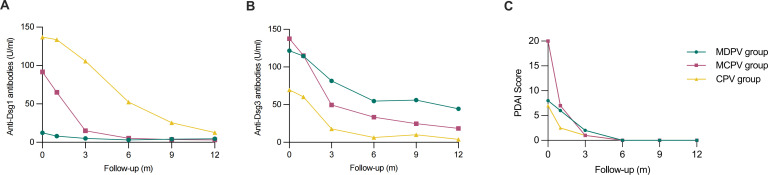
Trends in anti-Dsg1, anti-Dsg3 antibody titers and PDAI scores across different clinical subtypes in patients with PV during the 12-month period of follow up. **(A)** The Longitudinal changes of anti-Dsg1 antibody titers in the MDPV, MCPV, and CPV groups over time. **(B)** The trend of anti-Dsg3 antibody titers among the three groups throughout the follow-up. **(C)** PDAI scores declined in all groups during follow-up.

At disease onset, the highest anti-Dsg1 antibody titer was observed in the CPV group (136.95 U/ml), followed by MCPV (91.56 U/ml) and MDPV (12.37 U/ml). During follow-up, all groups exhibited a consistent decline in anti-Dsg1 titers, with CPV group maintaining the highest levels. Significant differences were found across time points within each group (*P*<0.05) (**Figure 1A**).

For anti-Dsg3 antibody titers, the MCPV group had the highest titer at baseline (137.64 U/ml), followed by MDPV (121.54 U/ml), while CPV group exhibited the lowest titer (69.56 U/ml). During follow-up, anti-Dsg3 antibody titers in MDPV and MCPV groups declined steadily, while in CPV group, anti-Dsg3 antibody titers decreased up to the 6-month follow-up, followed by a subsequent increase at 9-month follow-up (**Figure 1B**). Significant differences were noted across groups during follow-up (*P*<0.05) (**Figure 1B**).

At baseline, the MCPV group had the highest PDAI score (20), whereas MDPV and CPV groups scores of 8 and 7, respectively. PDAI scores decreased in all groups and stabilized after month 6, with significant differences (*P*<0.05) (**Figure 1C**).

Furthermore, correlation analysis between anti-Dsg1, anti-Dsg3 antibodies and disease activity was performed (**Table 2**). The results showed a positive correlation between anti-Dsg1, anti-Dsg3 antibodies and PDAI at baseline (*r*=0.368, *P*<0.001; *r*=0.321, *P*<0.001). No correlation was found at month 3, 6, 9. At 12 months, a weak correlation was observed between anti-Dsg1 and PDAI (*r*=0.322, *P*<0.001), whereas no correlation was found between anti-Dsg3 antibodies and PDAI.

**Table 2:** Correlation analysis between anti-Dsg1, anti-Dsg3 antibody titers and PDAI scores during follow-up.

Follow-up	Anti-Dsg1 versus PDAI		Anti-Dsg3 versus PDAI	
	*r*	*P* value	*r*	*P* value
Baseline	0.368	<0.001	0.321	<0.001
1 mo	0.268	<0.001	0.107	0.099
3 mo	0.229	<0.001	0.250	<0.001
6 mo	0.217	<0.001	0.237	0.001
9 mo	0.204	0.002	0.149	0.021
12 mo	0.322	<0.001	0.239	<0.001

Dsg, Desmoglein; PDAI, Pemphigus Disease Area Index.

### 3.3 Characteristics of persistent anti-Dsg antibody positivity in PV patients after CR

Among the 118 PV patients who achieved CR, we classified them into three groups based on antibody status (**Table 3**). Patients with persistent anti-Dsg1 positivity after CR had significantly higher baseline PDAI scores compared to the other two groups. Additionally, elevated baseline anti-Dsg3 levels were specifically observed in the group with persistent anti-Dsg3 positivity. Regarding affected sites, the majority of patients in the antibody-negative group exhibited both skin and mucosal involvement (82%), while patients with persistent anti-Dsg1 positivity were exclusively associated with combined skin and mucosal lesions.

**Table 3:** Characteristics of 118 PV patients achieving CR.

Characteristics	Anti-Dsg1/3 antibodies negative after CR (n=84)	Persistent anti-Dsg3 positivity after CR (n=32)	Persistent anti-Dsg1 positivity after CR (n=2)	*P*
Gender, n (%)				0.119
Male	32 (38)	19 (59)	1 (50)	
Female	52 (62)	13 (41)	1 (50)	
Age at diagnosis, median (IQR), year	49 (40-54)	45 (38-57)	46 (44-48)	0.976
Disease duration at baseline, Median (IQR), month	5 (3-12)	6 (3-18)	14 (4-24)	0.580
PDAI; M (IQR)	19 (6-29)	11 (2-19)	32 (28-36)	0.017
Disease severity				
Mild, n (%)	38 (45)	23 (72)	1 (50)	0.994
Moderate, n (%)	42 (50)	8 (25)	1 (50)	0.985
Severe, n (%)	4 (5)	1 (3)	0 (0)	0.949
Anti-Dsg1 at baseline, U/ml; M (IQR)	82.3 (18.3-149.0)	70.1 (8.1-138.4)	186.1 (178.6- 193.6)	0.113
Anti-Dsg3 at baseline, U/ml; M (IQR)	113.3 (59.0-147.9)	159.7 (125.0- 172.5)	127.1 (99.2-154.9)	0.001
Clinical presentation at diagnosis, n (%)				
MCPV	63 (75)	21 (65.6)	2 (100.0)	0.412
CPV	10 (12)	2 (6.3)	0 (0.0)	0.597
MDPV	11 (13)	9 (28.1)	0 (0.0)	0.129
Affected site, n (%)				
Skin only, n (%)	6 (7)	2 (6.3)	0 (0.0)	<0.001
Mucosa only, n (%)	9 (11)	7 (21.9)	0 (0.0)	0.252
Both skin and mucosa, n (%)	69 (82)	23 (71.9)	2 (100.0)	0.433

CR, complete Remission; IQR, interquartile range; PDAI, Pemphigus Disease Area Index; Dsg, desmoglein; MDPV, mucosal-dominant pemphigus vulgaris; MCPV, mucocutaneous pemphigus vulgaris; CPV, cutaneous pemphigus vulgaris.

Furthermore, 113 (95.7%) patients achieved clinical remission on minimal therapy (CRMT), while 5 (4.2%) patients achieved clinical remission off therapy (CROT) after 12 months of treatment. No significant differences in anti-Dsg1 and anti-Dsg3 positivity were observed between the two groups (*P >*0.05).

These findings suggest that higher baseline PDAI scores and elevated anti-Dsg3 antibody levels are associated with persistent antibody positivity, indicating a potential need for closer monitoring in these subgroups to achieve sustained remission.

### 3.4 Risk factors associated with persistent anti-Dsg1 antibody positivity after CR

Logistic regression analysis of factors related to persistent anti-Dsg1 antibody positivity after CR was detailed in **Table 4**. The results indicated that patients who developed cataracts as an adverse drug reaction had a significantly higher likelihood of persistent anti-Dsg1 antibody positivity (OR = 37.67; 95%CI: 1.88-756.27, *P* = 0.018). This suggests a potential link between this comorbidity and ongoing antibody presence.

**Table 4:** Univariable logistic regression model of factors associated with positive anti-Dsg1 and anti-Dsg3 antibodies after achieving CR.

Variable	Anti-Dsg1 antibody		Anti-Dsg3 antibody	
	OR (95%CI)	*P* value	OR (95%CI)	*P* value
Gender	1.28(0.08-20.87)	0.87	2.35(1.03-5.38)	0.044
Age	0.99(0.88-1.12)	0.90	1.00(0.97-1.04)	0.85
PDAI scores at baseline	1.08(0.97-1.19)	0.15	0.96(0.93-1.00)	0.024
Anti-Dsg1 at baseline	1.03(0.99-1.06)	0.13	1.00(0.99-1.00)	0.405
Anti-Dsg3 at baseline	1.00(0.98-1.03)	0.85	1.02(1.01-1.03)	0.001
Disease severity				
Mild	0.87(0.05-14.26)	0.92	0.99(0.44-2.22)	0.972
Moderate-severe	1.15(0.07-18.80)	0.92	1.02(0.45-2.29)	0.972
Treatment regimen	2.41(0.15-39.68)	0.54		
GC	0.59(0.04-9.67)	0.71	0.90(0.37-2.22)	0.824
GC combined with AZA	/	/	0.99(0.43-2.29)	0.977
GC combined with other IS	0	0.99	1.38(0.32-5.88)	0.664
RTX	1.00(0.91-1.09)	0.92	0.71(0.26-1.97)	0.516
Follow-up duration	1.02(0.93-1.12)	0.63	0.99(0.96-1.02)	0.348
Adverse reaction				
Cutaneous infection	/	0.99	0.47(0.15-1.51)	0.204
Fungal infection	8.67(0.51-147.68)	0.14	1.81(0.54-6.00)	0.335
Osteoporosis	4.52(0.27-75.28)	0.30	1.33(0.48-3.63)	0.583
Cataract	37.67(1.88-756.27)	0.018	0.89(0.09-8.91)	0.923

Dsg, Desmoglein; CR, Complete Remission; OR, Odds ratio; 95% CI, 95% Confidence Interval; PDAI, Pemphigus Disease Area Index; GC, Glucocorticoid; AZA, Azathioprine; IS, Immunosuppressant; RTX, Rituximab.

Additionally, further analysis was conducted in patients who developed cataracts. A total of 8 patients developed cataracts as an adverse effect, with ages ranging from 28 to 55 years. We performed Spearman rank coefficient analysis and no significant correlation between cataract development and age was found (r=-0.036; *P*=0.577).

### 3.5 Risk factors associated with persistent anti-Dsg3 antibody positivity after CR

Univariate analysis revealed that the strongest independent predictors for persistent anti-Dsg3 antibody positivity after CR were gender (OR = 2.35; 95%CI: 1.03-5.38, *P* = 0.044), PDAI scores at baseline (OR = 0.96; 95%CI: 0.93-1.00, *P* = 0.024) and anti- Dsg3 antibody titers at baseline (OR = 1.02; 95%CI: 1.01-1.03, *P* = 0.001) (**Table 4**). For further eliminating confounding influence, we included variables with a *p*-value < 0.05 in the univariate analysis to final multivariate model. The final independent predictors identified were gender (OR = 2.63; 95%CI: 1.01-6.85, *P* = 0.049), PDAI scores at baseline (OR = 0.93; 95%CI: 0.90-0.98, *P* = 0.002) and anti-Dsg3 antibody titers at baseline (OR = 1.02; 95%CI: 1.01-1.03, *P* < 0.001) (**Table 5**). These findings suggest that gender and specific baseline PDAI and anti-Dsg3 antibody titers may play a critical role in predicting the persistence of anti-Dsg3 antibodies in patients with PV following CR, which may have implications for ongoing monitoring and management strategies in this population.

**Table 5:** Multivariable logistic regression model of factors associated with positive anti-Dsg3 antibodies after achieving CR.

Variable	Multivariable model	
	OR (95%CI)	*P* value
Gender	2.63(1.01-6.85)	0.049
PDAI scores at baseline	0.93(0.90-0.98)	0.002
Anti-Dsg3 at baseline	1.02(1.01-1.03)	<0.001

Dsg3, Desmoglein3; CR, Complete Remission; OR, Odds ratio; 95%CI, 95% Confidence Interval; PDAI, Pemphigus Disease Area Index.

### 3.6 Cut-off values of anti-Dsg3 antibody

**Figure 2:**
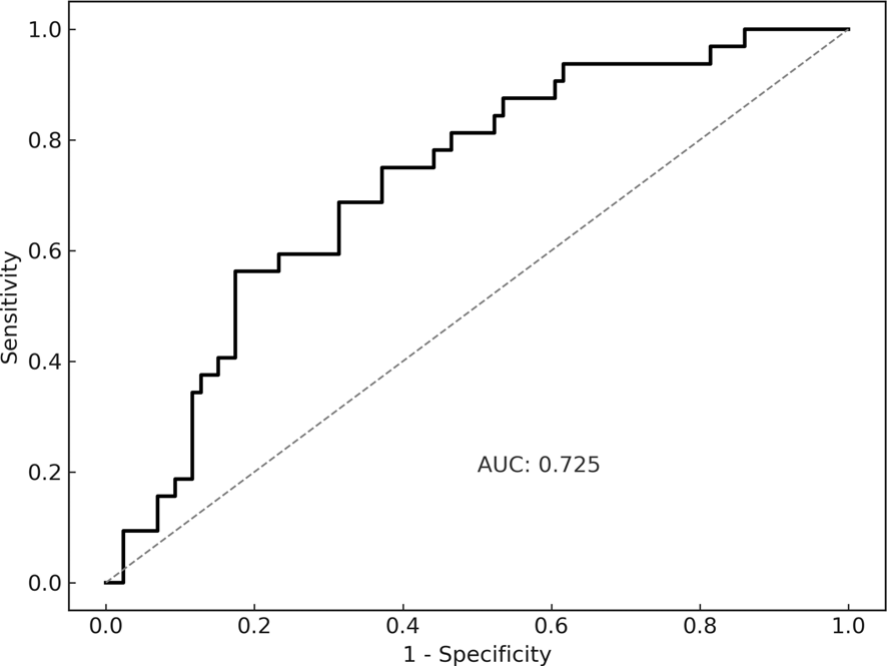
ROC curves showing the accuracy of anti-Dsg3 antibodies in predicting positive anti-Dsg3 antibodies after CR. (AUC = 0.725; sensitivity = 56.3%; specificity = 82.6%).

Based on our findings, we performed ROC analysis to determine the baseline anti-Dsg3 antibody cut-off value, identifying 157.4 U/mL as the optimal predictor with a sensitivity of 56.3% and specificity of 82.6% (**Figure 2**). This threshold is critical for the clinical assessment and management of anti-Dsg3 antibody positivity in PV patients after CR, helping identify the patients at higher risk of relapse.

## 4 Discussion

In this study, we presented a comprehensive analysis of demographic, clinical, and serological characteristics in patients with PV, with a focus on factors associated with persistent positivity of anti- Dsg1 and anti-Dsg3 antibodies after achieving CR. Our findings provide insights into the dynamic interplay between disease activity, antibody profiles, and clinical outcomes in PV, which have important implications for disease management and personalized care.

Previous studies have shown that the positivity rates of anti-Dsg antibodies vary across different ethnicities and regions. In India, 75% of PV patients detected positive for anti-Dsg1 antibodies, compared to 46% in Northern Europe (16). Similarly, a Japanese study reported the positivity rates for anti-Dsg1 and anti-Dsg3 antibodies were 59.2% and 88.3%, respectively (17). In our cohort, we found that 75% of PV patients were positive for anti-Dsg1, aligning closely with findings in Indian, whereas 94% were positive for anti-Dsg3, a higher rate than observed in other regions. These discrepancies highlight the potential influence of genetic, environmental, and lifestyle factors on antibody production and disease manifestation, warranting further investigation. Despite variations in treatment regimens, no statistically significant differences in anti-Dsg1 and anti-Dsg3 antibody positivity were found between patients treated with GC combined with IS and those treated with RTX. This lack of significant difference in autoantibody positivity suggests that the treatment regimen, whether involving RTX or GC combined with IS, may not have a direct impact on the persistence of anti-Dsg1 and anti-Dsg3 antibodies after treatment.

The Dsg compensation theory explains the close relationship between autoantibodies and clinical phenotypes in pemphigus (1). However, inconsistencies have been noted, as subsequent studies have found that additional antigens, such as Dsg2, Dsg4, desmocollin, desmoplakin, thyroid peroxidase, and type VII collagen may also contribute to the pathogenesis of PV (18–22). Some studies suggest that elevated anti-Dsg antibodies during remission may be due to the detection of non-pathogenic antibodies targeting calcium-dependent epitopes, such as monoclonal antibodies AK15 and AK18, or antibodies against EC1 and EC5. Other possible explanations include disease activity suppression from low-dose prednisone or adjuvant therapy (23). These findings indicate that the immune targets in PV are more diverse than previously thought, underscoring a need for a broader understanding of the molecular mechanisms underlying the disease. This expanded perspective may also partially explain why antibodies against Dsg1 and Dsg3 remain detectable during CR. In our study, anti-Dsg1 and anti-Dsg3 antibodies were positively correlated with PDAI scores at baseline (*P*<0.05), consistent with previous studies (24). However, no correlation was observed at month 3, 6 and 9. At the 12-month follow-up, a weak correlation was observed between anti-Dsg1 antibodies and PDAI, while no correlation was detected between anti-Dsg3 antibodies and PDAI. This suggests that compared to anti-Dsg3 antibodies, anti-Dsg1 antibodies have a relatively more significant impact on disease severity. Moreover, our study also found that some patients continued to have elevated levels of anti-Dsg antibodies even after achieving remission. This indicates that disease severity is not always directly related to anti-Dsg1, anti-Dsg3 antibody levels.

After 12 months of follow-up, we observed that a substantial proportion of the 118 patients who have achieved CR continued to exhibit antibody positivity. This persistent seropositivity suggests the presence of ongoing immune dysregulation, despite the absence of clinical symptoms. A retrospective cohort study using bead aggregation assays assessed the pathogenicity of elevated antibodies in clinical remission of PV patients. Results showed that 77.8% of sera in remission remained pathogenic, suggesting that the pathogenicity of anti-Dsg antibodies in pemphigus is primarily related to the titers of anti-Dsg antibodies in most patients (25). The lack of concordance between serological markers and clinical remission highlights the limitations of relying solely on serological assessments to evaluate disease condition.

The association between cataract development and persistent anti-Dsg1 positivity observed in our study is noteworthy. Cataracts are a well-established adverse effect of prolonged corticosteroid use. Based on etiology, cataracts can be classified as age-related, pediatric, and secondary type, with age-related cataract being the most common type, typically developing between the age of 45 and 50 years. Immunosuppressive diseases are also recognized as risk factors for cataract formation (26). In our cohort, 8 patients developed cataracts during follow-up, with ages ranging from 28 to 55 years but no significant correlation between age and cataract development was observed (r=-0.036, *P*=0.577), suggesting that age-related cataract alone may not fully explain this relationship.

While the direct link between anti-Dsg1 antibodies and cataract formation remains unclear, one potential explanation could be the underlying chronic inflammation and immune dysregulation in PV patients, which may contribute to both sustained antibody levels and cataract development. Furthermore, cumulative corticosteroid use may also play a role in cataract formation. And individual susceptibility to corticosteroid side effects might correlate with specific immune responses, which may induce persistent Dsg1 autoantibody production. Patients with PV and cataract may warrant closer monitoring for persistent antibody positivity, which could have implications for disease management and relapse risk assessment. Further mechanistic studies would be needed to explore these possibilities.

Our analysis also identifies independent predictors of persistent anti-Dsg3 positivity after CR, including elevated baseline anti-Dsg3 antibody titers, baseline PDAI scores and gender. These predictors can be instrumental in guiding clinical decision-making, particularly in identifying patients who may benefit from more intensive monitoring or early intervention to prevent relapse. For instance, patients with elevated baseline PDAI scores and anti-Dsg3 antibody titers may require closer follow-up and tailored treatment adjustments to achieve sustained remission. The identification of a specific cutoff value for anti-Dsg3 antibody levels that predicts the risk of persistent positivity further supports the clinical utility of these serological markers. This cutoff can serve as a reference for clinicians in assessing the risk of relapse and making informed decisions regarding the intensity and duration of immunosuppressive therapy.

This study has several limitations, including its single-center design, non-randomization allocation of patients, and limited follow-up duration, which may introduce selection bias and hinder long-term outcome observations. The duration of prior treatment was not fully characterized, which may have affected antibody levels. Additionally, the small number of patients with persistent anti-Dsg1 positivity may limit the strength of conclusions. Therefore, further multicenter, prospective studies with larger sample sizes and extended follow-up periods are needed to validate these findings.

In conclusion, our findings underscore the significance of anti-Dsg1 and anti-Dsg3 antibodies in the clinical management of PV. The identification of baseline disease severity, cataracts and specific antibody titer thresholds as predictors of disease course offers new avenues for monitoring and intervention. However, the observed discrepancies between antibody levels and clinical remission highlight the need for more comprehensive biomarkers that can guide treatment decisions more effectively. Future studies are needed to further elucidate the mechanisms underlying persistent antibody positivity and explore additional prognostic markers that can improve the precision of PV management.

## Data Availability

The original contributions presented in the study are included in the article/Supplementary Material. Further inquiries can be directed to the corresponding authors.
